# Modification of the Association between PM_10_ and Lung Function Decline by Cadherin 13 Polymorphisms in the SAPALDIA Cohort: A Genome-Wide Interaction Analysis

**DOI:** 10.1289/ehp.1307398

**Published:** 2014-08-15

**Authors:** Medea Imboden, Ashish Kumar, Ivan Curjuric, Martin Adam, Gian Andri Thun, Margot Haun, Ming-Yi Tsai, Marco Pons, Robert Bettschart, Alexander Turk, Thierry Rochat, Nino Künzli, Christian Schindler, Florian Kronenberg, Nicole M. Probst-Hensch

**Affiliations:** 1 Swiss Tropical and Public Health Institute, Basel, Switzerland; 2 University of Basel, Basel, Switzerland; 3 Wellcome Trust Centre for Human Genetics, University of Oxford, Oxford, United Kingdom; 4 Division of Genetic Epidemiology, Department of Medical Genetics, Molecular and Clinical Pharmacology, Innsbruck Medical University, Innsbruck, Austria; 5 Division of Pulmonary Medicine, Regional Hospital of Lugano, Lugano, Switzerland; 6 Lungenzentrum, Hirslanden Klinik, Aarau, Switzerland; 7 Zürcher Höhenklinik, Wald, Faltigberg-Wald, Switzerland; 8 Division of Pulmonary Medicine, University Hospitals of Geneva, Geneva, Switzerland

## Abstract

Background: Both air pollution and genetic variation have been shown to affect lung function. Their interaction has not been studied on a genome-wide scale to date.

Objectives: We aimed to identify, in an agnostic fashion, genes that modify the association between long-term air pollution exposure and annual lung function decline in an adult population-based sample.

Methods: A two-stage genome-wide interaction study was performed. The discovery (*n* = 763) and replication (*n* = 3,896) samples were derived from the multi-center SAPALDIA cohort (Swiss Cohort Study on Air Pollution and Lung Disease in Adults). Annual rate of decline in the forced mid-expiratory flow (FEF_25–75%_) was the main end point. Multivariate linear regression analyses were used to identify potential multiplicative interactions between genotypes and 11-year cumulative PM_10_ exposure.

Results: We identified a cluster of variants intronic to the *CDH13* gene as the only locus with genome-wide significant interactions. The strongest interaction was observed for rs2325934 (*p* = 8.8 × 10^–10^). Replication of the interaction between this *CDH13* variant and cumulative PM_10_ exposure on annual decline in FEF_25–75%_ was successful (*p* = 0.008). The interaction was not sensitive to adjustment for smoking or body weight.

Conclusions: *CDH13* is functionally linked to the adipokine adiponectin, an inflammatory regulator. Future studies need to confirm the interaction and assess how the result relates to previously observed interactions between air pollution and obesity on respiratory function.

Citation: Imboden M, Kumar A, Curjuric I, Adam M, Thun GA, Haun M, Tsai MY, Pons M, Bettschart R, Turk A, Rochat T, Künzli N, Schindler C, Kronenberg F, Probst-Hensch NM. 2015. Modification of the association between PM_10_ and lung function decline by cadherin 13 polymorphisms in the SAPALDIA cohort: a genome-wide interaction analysis. Environ Health Perspect 123:72–79; http://dx.doi.org/10.1289/ehp.1307398

## Introduction

Lung function is a complex phenotype influenced by lifestyle, environmental, and genetic factors. Inverse associations between chronic exposure to air pollution, such as particulate matter (PM), and respiratory function level as well as its decline have been reported in independent settings (Downs et al. 2007; Katanoda et al. 2011; Romieu et al. 2009; Schikowski et al. 2010). Air pollutants are thought to mediate their acute and chronic effects through an increase in oxidative stress, inflammation, and cytotoxicity (Andreau et al. 2012; Huang et al. 2012). However, mechanisms and differences in susceptibility remain poorly characterized (Brook et al. 2010). Only few candidate gene–air pollution interaction studies have been published. These reports also point to the oxidative and inflammatory effects of air pollution in mediating adverse respiratory health effects (Breton et al. 2011; Curjuric et al. 2012; Imboden et al. 2009; Melén et al. 2008; Romieu et al. 2006; Yang et al. 2005).

Genome-wide association studies (GWAS) on lung function were mostly cross-sectional in nature (Artigas et al. 2011; Hancock et al. 2010, 2012; Obeidat et al. 2011; Repapi et al. 2010; Wilk et al. 2009), and more recent reports have shown that the overlap in genetic determinants of the level of lung function and its decline is minimal (Hansel et al. 2013; Imboden et al. 2012). None of these lung function GWAS studies has considered ambient air pollution.

In the present study, we used a genome-wide interaction study (GWIS) approach to uncover novel genetic loci modifying the association between particulate matter exposure and 11-year lung function decline. We applied a two-stage approach with a discovery sample (*n* = 763) and a replication sample (*n* = 3,896). Both are subpopulations of the SAPALDIA cohort study (Swiss Cohort Study on Air Pollution And Lung Diseases In Adults) (Martin et al. 1997). This multi-center population-based cohort was specifically designed to investigate long-term effects of air pollution on respiratory health. We *a priori* chose forced mid-expiratory flow (FEF_25–75%_) as the dependant lung function phenotype because it was the outcome most strongly associated with ambient particulate matter air pollution exposure in SAPALDIA (Downs et al. 2007). As a proxy for long-term exposure to complex air pollution mixtures, we chose personal estimates of 11-year cumulative exposure to home outdoor PM mass with ≤ 10 μm in aerodynamic diameter (PM_10_) (Curjuric et al. 2012; Liu et al. 2007).

## Methods

*SAPALDIA cohort study*. SAPALDIA was initiated in 1991. Participants, 18–60 years of age, were randomly selected from the population registries of eight geographic Swiss regions, with varying degrees of urbanization and different environmental and cultural characteristics. Participants of the baseline examination (*n* = 9,651) were invited in 2002 (*n* = 8,047) for a second examination. Ethical approval was obtained from the Swiss Academy of Medical Sciences and the Regional Ethics Committees; written informed consent was obtained from all participants before health examination and biological sample collection at each survey. Study design and data collected have been described elsewhere (Ackermann-Liebrich et al. 2005). Briefly, health examinations and standardized questionnaires focused on respiratory and cardiovascular health. Formal collection of fractioned blood and DNA samples was established at the follow-up survey.

*Study population*. Nonparticipation at follow-up (*n* = 1,604) and missing information on lung function phenotype data (*n* = 2,302), genotype data (*n* = 476), or covariates (*n* = 43) led to the exclusion of some SAPALDIA cohort participants from the present study. Participants reporting a history of asthma were excluded (*n* = 567) because of evidence of genetic heterogeneity of lung function decline in asthmatic and nonasthmatic subjects (Imboden et al. 2012). The final study population included participants with blood samples available for genetic testing and complete baseline and follow-up data on spirometry, smoking history, weight, weight change, height, PM_10_ exposure, and residential history, as well as statistical model covariates (*n* = 4,659) (Downs et al. 2007). The discovery sample with genome-wide data was a random sample of the nonasthmatic SAPALDIA study population (*n* = 763) (Moffatt et al. 2010). The replication sample consisted of the remainder of SAPALDIA participants with complete data (*n* = 3,896) and was subjected to targeted genotyping for replication of promising discovery interaction signals.

*Phenotype and covariate assessment*. For lung function assessment, identical spirometer devices (Sensormedics model 2200; Sensormedics, Yorba Linda, CA, USA) and protocols were used at both examinations (Ackermann-Liebrich et al. 2005). Comparability of devices was ascertained (Künzli et al. 2005). Each participant performed three to a maximum of eight forced expiratory lung function maneuvers to obtain a minimum of two acceptable forced expiratory flows, forced vital capacity (FVC), forced expiratory volume in the first second (FEV_1_) complying with American Thoracic Society (1995) criteria. Expiratory flow measures during the middle half of the FVC (FEF_25–75%_) were taken from the flow-volume curves with the highest sum of FVC and FEV_1_. Given evidence from the SAPALDIA cohort, we focused in the present study on the annual rate of decline in FEF_25–75%_ as a sensitive marker of age-related decline because it was more strongly associated with the PM_10_ exposure than was FEV_1_ or FEV_1_/FVC decline (Curjuric et al. 2010; Downs et al. 2007; Imboden et al. 2009; Thun et al. 2012). Annual decline in FEF_25–75%_ was calculated as the difference between follow-up and baseline measure in milliters per second, divided by length of follow-up in years. Accordingly, declines in FEV_1_, FEV_1_/FVC, and FEF_25–75%_/FVC were calculated for sensitivity analyses of the FEF_25–75%_ GWIS top hits. Covariate information was assessed including a computer-assisted personal interview at baseline and follow-up examinations, including age, sex, current and past smoking status, and smoking history (number of cigarettes/day, years of smoking). The exposure to other inhaled pollutants such as environmental tobacco smoke or occupational exposure to dust and fumes, and respiratory symptoms were assessed with the same questions at both surveys. Participants who reported smoking < 20 packs of cigarettes and < 360 g of tobacco in their lifetime at both time points were defined as never-smokers. Cumulative cigarette exposure of participants was assessed by pack-years smoked before the first examination and pack-years smoked during follow-up. Height was measured (without shoes) at baseline and follow-up. Weight was self-reported at baseline and measured at follow-up (without shoes and coat). Weight change was calculated as weight at follow-up minus weight at baseline, with positive values reflecting weight gain during follow-up period.

*Home outdoor PM_10_ exposure assessment*. We used PM_10_ as the air pollution exposure measure. Air pollution exposure assessment, dispersion model validation, as well as attribution of individual 11-year cumulative PM_10_ exposure have been described elsewhere (Liu et al. 2007). Briefly, a hybrid exposure model incorporated geocoded data on seasonal, meteorological, and traffic, industrial, regional, and agricultural emission activities. Hourly concentrations of PM_10_ were calculated on a spatial resolution of 200 × 200 m grid cells over the follow-up period. Annual averages of the modeled PM_10_ concentrations were obtained for each grid cell. We estimated the cumulative PM_10_ exposure for study participants using their residential history, in geocoded data format, assigning annual PM_10_ exposure averages derived from the grid cells generated by the dispersion model, and adding up the averages over the 11-year follow-up period (Liu et al. 2007).

*Genotyping, imputation, population stratification, and quality control*. DNA extraction from EDTA-buffered whole blood has been previously described (Ackermann-Liebrich et al. 2005; Imboden et al. 2006). Genome-wide genotyping was obtained using the platform Illumina 610K quad Bead Chip. Discovery genotyping quality control, imputation, and correction for population stratification was applied as previously described (Moffatt et al. 2010). Briefly, genome-wide genotyping was centrally performed for the GABRIEL asthma Consortium at the Centre National de Génotypage (CNG, Evry, France). We satisfactorily genotyped 567,589 autosomal single nucleotide polymorphisms (SNPs) (mean call rate, 99.7%). We obtained 2,588,592 autosomal HapMap-based SNPs by cohort-specific imputation using the MACH v1.00 software and the HapMap2 release 22 CEU reference sample (Moffatt et al. 2010). Statistical power to detect gene–environment interaction is expected to be limited, so we excluded SNPs with minor allele frequencies < 5% to avoid inflation of false positive findings produced by rare genetic variants. Final number of SNPs used for interaction association testing was 2,198,793. To account for population stratification, we relied on inferred ancestry-informative principal components (Moffatt et al. 2010) that were previously carried out using EIGENSTRAT 2.0 software and the all ethnicity HapMap data, as well as additional European reference samples (Heath et al. 2008). Subjects of non-European descent were excluded based on the first and second principal components. Adjustment for population stratification in the linear regression analyses was done by incorporating the third and fourth principal component in the statistical model. Cryptic relatedness was detected based on identity-by-state analysis, and one participant per family cluster was retained in the study population.

*Statistical analysis*. Discovery sample. We performed agnostic GWIS analysis in the discovery sample using an additive genetic model, with *a priori* selection of potential confounders based on previous analyses of the association between air pollution and lung function decline (Curjuric et al. 2010; Downs et al. 2007; Imboden et al. 2009). We regressed each SNP with cumulative PM_10_ on FEF_25–75%_ annual decline adjusting for study center, age, sex, height, never-smoking status, seasonal effects (sine and cosine function of day of examination), and population stratification factors. In addition, models were adjusted for weight at baseline, weight change during follow-up, and the multiplicative interaction between baseline weight and weight change, based on our recent analysis demonstrating an interaction between air pollution and obesity on lung function in the study population (Schikowski et al. 2013). Potential interaction effects between genotype and PM_10_ were captured by the inclusion of a multiplicative interaction term in the linear multivariate regression analyses. We used a joint test with two degrees of freedom to derive *p*-values for the joint effects of gene marginal and gene–environment interactions. This approach has been shown under a range of scenarios to have greater power for identifying novel genetic candidates than tests of the gene marginal effect or gene–environment interaction effects alone (Hancock et al. 2012; Kraft et al. 2007). We used the following terminology to report the results of the genetic effects related to the gene marginal (*p*_main_), the gene–environment (*p*_int_), and the joint (*p*_joint_) effects referring to their respective null hypothesis of gene marginal (β_main_ = 0), the gene–environment (β_int_ = 0), and the joint (β_main_ = 0 and β_int_ = 0) effects. We defined the genome-wide significance level at *p* < 5 × 10^–8^ using Bonferroni adjustment for one million independent tests. The lambda (λ) for the main GWIS—a metric for estimating genomic inflation of the observed associations—was calculated as the ratio of the observed versus expected median of the chi-square distribution with 2 degrees of freedom [ΣChi2(2df); median_observed_ divided by 1.386 (median_expected_)]. GWIS sensitivity analyses were additionally performed on FEV_1_, FEV_1_/FVC, and FEF_25–75%_/FVC decline to determine the genome-wide ranking of the FEF_25–75%_ top hits in the GWIS results for other lung function phenotypes.

Replication sample. The replication analyses was performed on two intronic *CDH13* SNPs exhibiting the lowest or very low *p*-values in the discovery phase without being in high linkage disequilibrium with one other (top hit, rs232593, and rs17284098). Replication *de novo* genotyping of rs2325934 and rs17284098 was performed on a 7900HT Fast Real-Time PCR System (Applied Biosystems, Foster City, CA, USA) by using 5´-nuclease allelic discrimination assays. A random sample of approximately 10% of all DNA samples was re-genotyped, and all genotypes were confirmed. The genotype call rate was > 99%. The same adjustments as for the discovery GWIS were used, except that adjustment for population stratification was not possible in the replication sample. We do not, however, expect associations to be confounded in the replication sample because adjustment for population stratification did not influence associations in the discovery sample (data not shown). Given the gene–environment interaction identified, we performed genotype stratified analyses in the combined sample (discovery and replication) as well as additional explorative analyses assessing the robustness of the observed PM_10_–*CDH13* interaction with a particular focus on smoking and on weight-related variables.

*Post hoc analysis on* CDH13. In a post hoc analysis, we first searched the dbGaP database (http://www.ncbi.nlm.nih.gov/projects/gapplusprev/sgap_plus.htm) for reported associations of *CDH13* genetic variants using “CDH13” as the search term and looked up the GWIS result of these SNPs for interaction with PM_10_ on decline in FEF_25–75%_. Second, we used the imputed data obtained in the SAPALDIA discovery sample to construct haplotypes in a 200-kb chromosomal window centered on the GWIS top hit using the software Haploview (Barrett et al. 2005). Third, based on the strong functional link between *CDH13* and adiponectin, we looked up the GWIS result of SNPs in the *ADIPOQ* gene, the adiponectin precursor protein, for interaction with PM_10_ on decline in FEF_25–75%_ and made a regional association plot of the *ADIPOQ* locus using the software LocusZoom (Pruim et al. 2010).

## Results

Baseline characteristics of the SAPALDIA cohort study participants included in the current GWIS analysis of the discovery and replication sample are presented in Table 1. We observed a highly comparable distribution of sex, age, baseline body mass index, weight change during follow-up, baseline lung function level, and average PM_10_ exposure at baseline and during follow-up, except for a small difference in proportion in smokers and smoking intensity between the two samples (Table 1).

**Table 1:** Baseline characteristics of the study population of the SAPALDIA cohort, discovery and replication sample (mean ± SD or %).

Characteristic	Discovery	Replication
*n*	763	3,896
Female (%)	51.1	51.6
Age (years)	41.6 ± 11.0	41.1 ± 11.4
Body mass index (kg/m^2^)	23.5 ± 3.5	23.7 ± 3.6
Height (cm)	169.6 ± 9.0	169.5 ± 8.7
Weight (kg)	67.9 ± 12.6	68.4 ± 12.6
Weight change^*a*^ (kg)	5.4 ± 6.2	5.6 ± 6.0
Baseline lung function
FEF_25–75__%_ (L/sec)	3.5 ± 1.2	3.5 ± 1.2
FEV_1_ (L)	3.6 ± 0.8	3.6 ± 0.8
FEV_1_/FVC (%)	79.6 ± 7.0	79.4 ± 7.2
FEF_25–75%_/FVC (%)	78.1 ± 24.5	77.6 ± 24.7
Air pollution exposure
PM_10_ annual mean (μg/m^3^)	27.4 ± 9.4	27.3 ± 9.7
PM_10_ cumulative^*a*^ (μg/m^3^)	246.5 ± 79.0	245.2 ± 81.4
Smoking status
Never-smokers^*b*^ (%)	42.9	43.8
Pack-years^*c*^	16.5 ± 17.3	17.0 ± 18.2
Pack-years^*a,c*^	5.4 ± 6.5	6.3 ± 8.3
Abbreviations: FEF_25–75%,_ forced mid-expiratory flow; FEF_25–75%_/FVC, ratio of forced mid-expiratory flow and of forced vital capacity; FEV_1,_ forced expiratory volume in the first second; FEV_1_/FVC, ratio of forced expiratory volume in the first second and of forced vital capacity; FVC, forced vital capacity. ^***a***^During 11-year follow-up. ^***b***^Never-smokers defined as nonsmoker at baseline and at follow-up survey. ^***c***^Discovery sample: missing data on pack-years at baseline (*n *= 20, 2.6%) and during follow-up (*n *= 62, 7.3%); replication sample: missing data on pack-years at baseline (*n *= 67, 1.7%) and during follow-up (*n *= 326, 8.4%).		

**Figure 1:**
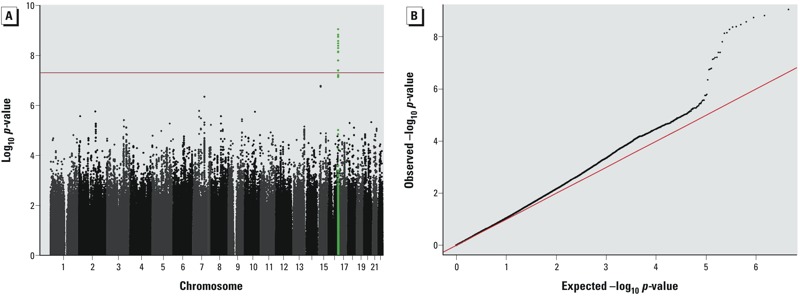
Genome-wide interactions between cumulative PM_10_ exposure on annual decline in FEF_25–75%_ in the discovery sample (*n *= 763) of the SAPALDIA cohort study. (*A*) Manhattan plot of the negative log of the *p*-values (*p*_int_) of 2,198,793 SNPs used for interaction association testing. *CDH13* SNP cluster with interaction *p*-values reaching genome-wide significance are above the line and SNPs in the *CDH13* locus are highlighted in green. (*B*) Quantile–quantile plot representing calculated *p*-values (*p*_int_) for each PM_10_ by SNP interaction tested plotted against the expected chi-square–distributed *p*-values. Deviation from the diagonal identity line points to the presence of potentially true associations.

*GWIS discovery results*. We observed significant association signals with a group of 13 SNPs, interacting with cumulative PM_10_ exposure on annual decline in FEF_25–75%_, at a single locus on chromosome 16 (Figure 1A). The quantile–quantile plot of interaction *p*-values showed evidence for a higher number of significant signals than expected by chance (Figure 1B). Based on the lambda observed (λ = 1.0476), adequate genomic control of the genome-wide associations had been applied.

**Figure 2:**
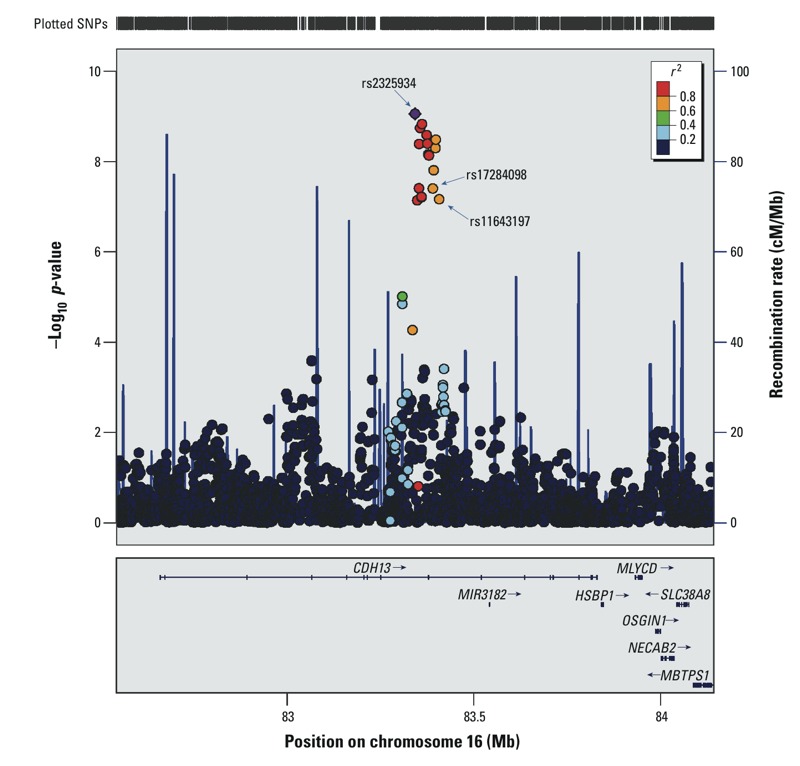
Regional association plot showing the *p*-values of interaction between cumulative PM_10_ exposure and *CDH13* SNPs on annual decline in FEF_25–75%_ in the discovery sample (*n *= 763) of the SAPALDIA cohort study. Shown is the regional association plot for the genome-wide significant GWIS association signal located in the *CDH13* gene at 16q23.3. Negative log of the *p*-values are plotted on the *y*-axis. Genomic coordinates (Mb) of the plotted SNPs refer to genome build 36/hg18 and dbSNP128 and are given on the *x*-axis. Linkage disequilibrium information (*r*^2^ values) refers to HapMap Phase II data of Caucasian samples. Recombination rate shown over this chromosomal window indicates recombination sites as vertical lines. The plot was generated using LocusZoom (Pruim et al. 2010). Genes in the genomic vicinity are *HSBP1,* heat-shock factor-binding protein 1; *MBTPS1,* membrane-bound transcription factor protease, site 1; *MIR3182,* microRNA 3182; *MLYCD,* malonyl-CoA decarboxylase; *NECAB2,* N-terminal EF-hand calcium binding protein 2; *OSGIN1, *oxidative stress-induced growth inhibitor 1; *SLC38A8,* solute carrier family 38 (amino acid transporter), member 8.

The association signal at 16q23.3, located intronic to gene *CDH13* (Table 2), had the strongest interaction (*p*_int_ = 8.8 × 10^–10^) for rs2325934, an uncommon variant [MAF (frequency of the least common allele in the study sample), 9.6%]. There was evidence for additional potentially independent interaction signals in this locus, as variants with varying MAFs and differing linkage disequilibrium (LD) values also showed significant interactions with cumulative PM_10_ on annual FEF_25–75%_ decline (e.g., rs11643197: MAF, 13.4%, *p*_int_ = 6.87 × 10^–8^, LD with the *CDH13* top hit rs2325934 *r*^2^ = 0.585 and D´ = 1) (Figure 2; see also Supplemental Material, Figure S1). *p*-Values for gene main effects (*p*_main_), gene–environment interaction effects (*p*_int_), and the joint test (*p*_joint_) are provided in Table 2 for intronic *CHD13* SNPs, and in Supplemental Material, Table S1, for the top 1,000 SNPs associated with the decline in FEF_25–75%_ in the discovery GWIS, ranked by gene–environment interaction effects (*p*_int_). The joint test of the SNP main effect and the interaction effect (*p*_joint_, null hypothesis: β_main_ = 0 and β_int_ = 0) did not identify additional genetic modifiers of the association between cumulative PM_10_ and lung function decline beyond those already identified based on *p*_int_ for the gene–environment interaction, and the *CDH13* variants ranked high according to gene main effect, interaction, and joint tests (maximum genome-wide rank = 22 for the joint test; Table 2).

**Table 2:** Discovery GWIS top hits with *p*-values for interaction (*p*_int_) < 10^–7^, clustered intronically to the *CDH13* gene: adjusted interaction association with individualized cumulative PM_10_ exposure on annual decline in FEF_25–75%_ in the SAPALDIA cohort.

dbSNP ID	Chromosome	Position	Minor allele frequency (%)	Main*p*_main_	Interaction*p*_int_	Joint*p*_joint_	Genome-wide test rank			Strong LD groups^*a*^
							Main	Interaction	Joint	
rs2325934^*b*^	16	81900000	9.64	6.94 × 10^–11^	8.80 × 10^–10^	5.80 × 10^–10^	1	1	1	Reference (A)
rs17282232	16	81905824	11.12	5.26 × 10^–9^	7.28 × 10^–8^	3.64 × 10^–8^	13	17	13	Reference (B)
rs10514582	16	81910432	8.66	3.71 × 10^–9^	3.93 × 10^–8^	2.72 × 10^–8^	11	12	11	A
rs10514580	16	81910872	9.71	1.89 × 10^–10^	4.10 × 10^–9^	1.47 × 10^–9^	4	7	4	A
rs16960234	16	81913512	9.95	9.90 × 10^–11^	1.82 × 10^–9^	8.05 × 10^–10^	2	3	2	A
rs12325503	16	81917248	11.21	5.98 × 10^–9^	6.23 × 10^–8^	4.30 × 10^–8^	14	15	14	B
rs10514578	16	81917312	11.20	6.01 × 10^–9^	6.07 × 10^–8^	4.34 × 10^–8^	15	14	15	B
rs17210599	16	81918568	9.93	1.18 × 10^–10^	1.49 × 10^–9^	9.77 × 10^–10^	3	2	3	A
rs10514575	16	81931320	9.85	3.95 × 10^–10^	2.61 × 10^–9^	3.12 × 10^–9^	5	4	5	A
rs17211371	16	81933040	9.98	7.20 × 10^–10^	4.03 × 10^–9^	5.56 × 10^–9^	6	6	6	A
rs1424168	16	81935600	10.10	1.36 × 10^–9^	6.95 × 10^–9^	1.03 × 10^–8^	7	9	7	A
rs17211581	16	81937240	10.11	1.44 × 10^–9^	7.40 × 10^–9^	1.09 × 10^–8^	8	10	9	A
rs17284098^*b*^	16	81947576	12.87	1.56 × 10^–8^	3.98 × 10^–8^	1.02 × 10^–7^	16	13	16	Reference (C)
rs17284265	16	81949792	12.14	5.13 × 10^–9^	1.57 × 10^–8^	3.55 × 10^–8^	12	11	12	C
rs17284390	16	81954784	11.90	2.01 × 10^–9^	5.08 × 10^–9^	1.33 × 10^–8^	10	8	10	C
rs17212165	16	81955688	11.96	1.71 × 10^–9^	3.30 × 10^–9^	1.05 × 10^–8^	9	5	8	C
rs11643197	16	81964792	13.36	6.35 × 10^–8^	6.87 × 10^–8^	3.17 × 10^–7^	17	16	22	C
Ranks shown in the table refer to the genome-wide ranking over 2,198,793 SNPs. The following terminology defined genetic effects referring to their respective null hypothesis: *p*_main_: gene marginal (β_main_ = 0); *p*_int_: gene–environment (β_int_ = 0); *p*_joint_: joint effect (β_main_ = 0 and β_int_ = 0).^******^GWIS was adjusted for study center, age, sex, height, never-smoking status, weight at baseline, weight change during follow-up, interaction between baseline weight and weight change, seasonal effects of time point of baseline and follow-up examination date (sine and cosine function of day of examination) and population stratification components. Cohort participants with self-report of asthma history had been excluded from the analysis. Discovery sample size was *n *= 763. ^***a***^LD (linkage disequilibrium): A indicates strong LD with rs2325934 (*r*^2^ > 0.85; D´ = 1), B indicates strong LD with rs17282232 (*r*^2^ > 0.85; D´ = 1), and C indicates strong LD with rs17284098 (*r*^2^ > 0.85; D´ = 1). The replication SNPs, rs2325934 and rs17284098, were in the moderate linkage disequilibrium with each other (*r*^2^ = 0.685, D´ = 1). ^***b***^SNPs selected for replication; they were in the moderate linkage disequilibrium with respect to *r*^2^ (*r*^2^ = 0.685, D´ = 1).										

Next, we performed sensitivity GWIS analyses for annual decline in FEV_1_, in FEV_1_/FVC, and in FEF_25–75%_/FVC to assess the ranking of the *CDH13* variants (see Supplemental Material, Table S2). Briefly, although genome-wide significance was not reached, the *CDH13* locus was the top-ranking locus in the GWIS for decline in ratios of both FEV_1_/FVC and FEF_25–75%_/FVC, but it was not strongly associated with annual decline in FEV_1_ (*p*_int_ ≥ 0.001). For FEV_1_/FVC and FEF_25–75%_/FVC, the *CDH13* SNP with the strongest interaction *p*-value was rs2325934 (for FEV_1_/FVC: *p*_int_ = 1.99 × 10^–6^; for FEF_25–75%_/FVC: *p*_int_ = 1.47 × 10^–6^).

*GWIS replication results*. We selected two genome-wide significant *CDH13* SNPs for *de novo* genotyping in the replication study sample (*n* = 3,896). The rs2325934 variant was selected because it exhibited the lowest *p*-value of association (*p*_int_) in the discovery analysis. A second SNP, rs17284098 (MAF, 12.9%; discovery *p*_int_ = 3.98 × 10^–8^) was chosen for its higher MAF compared with the top hit. Both replication SNPs were in moderate LD (*r*^2^ = 0.685 and D´ = 1). They replicated yielding *p*-values below the Bonferroni corrected significance level for two tests (*p* < 0.025; rs2325934: *p*_int_ = 0.008; rs17284098: *p*_int_ = 0.016; Table 3). Interactions between the *CDH13* replication SNPs and PM_10_ were robust to adjustment for different covariates (Table 3). The observed association became slightly stronger despite diminished sample size (*n* = 3,504) when adjusted for history of smoking intensity, including pack-years at baseline and pack-years smoked during follow-up. However, it is not possible to determine whether the change was attributable to adjustment or to a difference in the sample. Omitting smoking adjustment resulted in weakened signal, as did omitting the interaction term between weight and weight change. In contrast, adjusting additionally for age-squared slightly strengthened the associations. The PM_10_ effect modification by the *CDH13* SNPs remained significant even in minimally adjusted (age, sex, and study area) models (rs2325934: *p*_int_ = 0.019; rs17284098: *p*_int_ = 0.020; Table 3).

**Table 3:** Replication results of adjusted*^a^* interaction of *CDH13* intronic SNPs (rs2325934 and rs17284098) with cumulative PM_10_ during 11-year follow-up on annual decline in FEF_25–75%_, the SAPALDIA cohort study.

dbSNP ID	*n*	Coefficient^*b*^ (95% CI)	SE	*p*_int_
Adjusted model^*a*^
rs2325934	3,879	0.0742 (0.0191, 0.1294)	0.0281	0.008
rs17284098	3,878	0.0632 (0.0117, 0.1147)	0.0263	0.016
Adjusted + smoking history^*c*^
rs2325934	3,504	0.0766 (0.0198, 0.1335)	0.029	0.008
rs17284098	3,504	0.0723 (0.0188, 0.1257)	0.0273	0.008
Adjusted + age-squared^*c*^
rs2325934	3,879	0.0755 (0.0205, 0.1306)	0.0281	0.007
rs17284098	3,878	0.0645 (0.0130, 0.1160)	0.0263	0.014
Adjusted: smoking status^*d*^
rs2325934	3,879	0.074 (0.0188, 0.1291)	0.0281	0.009
rs17284098	3,878	0.0632 (0.0118, 0.1147)	0.0262	0.016
Adjusted: interaction between baseline weight and weight change^*d*^
rs2325934	3,879	0.0742 (0.0191, 0.1293)	0.0281	0.008
rs17284098	3,878	0.0629 (0.0114, 0.1144)	0.0263	0.017
Adjusted: weight change and interaction between baseline weight and weight change^*d*^
rs2325934	3,879	0.0757 (0.0212, 0.1301)	0.0278	0.006
rs17284098	3,878	0.0626 (0.0116, 0.1136)	0.026	0.016
Adjusted: weight at baseline and interaction between baseline weight and weight change^*d*^
rs2325934	3,879	0.0766 (0.0211, 0.1320)	0.0283	0.007
rs17284098	3,878	0.0657 (0.0141, 0.1173)	0.0263	0.013
Minimal adjustment^*e*^
rs2325934	3,879	0.0659 (0.0108, 0.1211)	0.0282	0.019
rs17284098	3,878	0.0606 (0.0095, 0.1117)	0.0261	0.02
^***a***^Same adjustments applied as for discovery GWIS, including study center, age, sex, height, never-smoking status, weight at baseline, weight change during follow-up, interaction between baseline weight and weight change, seasonal effects of time point of baseline and follow-up examination date (sine and cosine function of day of examination). No adjustment for population stratification was available. ^***b***^Coefficient refers to the additive SNP effect in annual change in FEF_25–75%_ (mL/sec) per 1-μg/m^3^ change in PM_10_ exposure. ^***c***^Same adjustments as for the discovery GWIS, adding indicated additional covariate(s) in the model. ^***d***^Same adjustments as for the discovery GWIS, omitting indicated covariate(s) in the model. ^***e***^Basic adjustment including only study center, age, and sex in the model.				

In a genotype-stratified analysis, combining discovery and replication sample, for both *CDH13* SNPs, the PM_10_–FEF_25–75%_ association appeared to be restricted to participants who were homozygous for the major allele (see Supplemental Material, Table S3). For rs2325934, the major homozygous genotype strata (*n* = 3,750) was estimated to have an average annual change in FEF_25–75%_ of –0.102 mL per increase of 1 μg/m^3^ PM_10_ [95% confidence interval (CI): –0.19, –0.01; *p* = 0.03] in contrast with the group carrying at least one minor allele (estimated average annual change of 0.074 mL; 95% CI: –0.16, 0.31; *p* = 0.53, *n* = 886).

*Previously published* CDH13 *genome-wide association results and linkage disequilibrium in the 200-kb GWIS window.* In GWAS, variants of the *CDH13* gene were previously associated with a number of different phenotypes (see Supplemental Material, Table S4). Genetic variants in the 5´ end of the *CDH13* gene have been repeatedly associated with circulating adiponectin levels (Chung et al. 2011; Dastani et al. 2012; Jee et al. 2010; Morisaki et al. 2012; Wu et al. 2010). We thus looked up the interactions of these previous *CDH13* GWAS hits with PM_10_ on decline in FEF_25–75%_, but none of these SNPs ranked high in the current GWIS (*p*_int_ ≥ 0.02; see Supplemental Material, Table S4). Pairwise LD in the *CDH13* gene between the GWAS SNPs and the PM_10_ interacting SNPs was low (*r*^2^ ≤ 0.33).

The structure of linkage disequilibrium (see Supplemental Material, Figure S1) and haplotypes (see Supplemental Material, Figure S2) in the 200-kb window centered on rs2325934 were constructed using the imputed genotype data of the discovery sample. The LD pattern and derived haplotypes suggested that the GWIS top hit tagged specifically one 33 kb–long haplotype (block 9, stretching from rs2352934 to rs1426166). The second replication variant, rs17284098, was located in a different 11 kb–long haplotype (block 11, stretching from rs1424168 to rs17284098) in an intron downstream of the GWIS top hit.

## Discussion

To our knowledge, this is the first report presenting a genome-wide interaction study aiming to identify novel genes modifying the association of PM on lung function decline. We identified a cluster of SNPs intronic to the gene *CDH13* that modified the estimated effect of cumulative PM_10_ on the decline in FEF_25–75%_ in our study population. We estimated that participants who were homozygous for the major allele of rs2325934 experienced an excess average decline of 11 mL/sec in FEF_25–75%_ per 10-μg/m^3^ increase in cumulative PM_10_ exposure over 11 years. Interestingly, cadherin 13, the protein encoded by *CDH13* is functionally linked to adiponectin, a predominantly anti-inflammatory adipokine.

Experimental animal studies have provided strong evidence that major cellular responses to PM exposure include oxidative stress (Manzo et al. 2012) and inflammation (Uski et al. 2012). Results from 125 subjects monitored before, during, and after the Beijing Olympics were consistent with oxidative and inflammatory effects of ambient air pollution in the respiratory tract (Huang et al. 2012). The relationship between PM exposure and systemic inflammation, as indicated by serum C-reactive protein (CRP), was the subject of a recent systematic review reporting more consistent results for a positive PM–CRP association in longitudinal studies of healthy subjects than in short-term studies or longitudinal studies of subjects with chronic inflammatory conditions (Li et al. 2012). The few candidate gene–air pollution interaction studies published to date also support the oxidative and inflammatory effects of air pollution in mediating adverse respiratory health effects (Breton et al. 2011; Curjuric et al. 2010; Imboden et al. 2009; Melén et al. 2008; Romieu et al. 2006; Yang et al. 2005).

**Figure 3:**
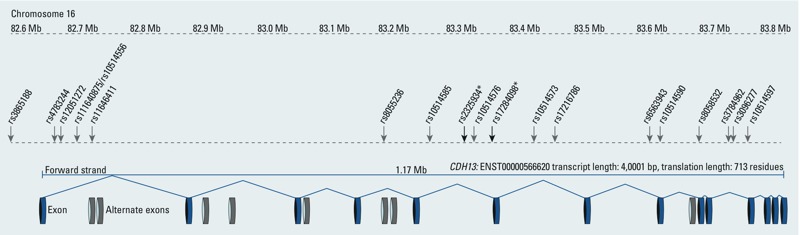
Schematic representation of *CDH13* gene and genetic variants identified by GWAS to be associated with various phenotypes. The *CDH13* gene, spanning 1.17 Mb, at 82.6 Mb (build 36) on chromosome 16, encodes 15 different transcripts with alternate exons which produce structural proteins. The major *CDH13* transcript contains 14 exons, encoding an open reading frame for a 713-amino acid polypeptide. In this schematic view of the *CDH13* gene, we pinpoint SNPs identified in the dbGaP database (http://www.ncbi.nlm.nih.gov/projects/gapplusprev/sgap_plus.htm) for reported associations with various phenotypes using “CDH13” as the search term. For a list of the associated phenotypes, see Supplemental Material, Table S4. *SNPs, rs2325934 and rs17284098, identified in the present report to interact with PM_10_ exposure on decline in FEF_25–75%_.

In humans, *CDH13* is expressed in various lung cell types, including bronchial epithelium and airway smooth muscle cells. The *CDH13* gene, spanning 1.17 Mb, encodes 15 different transcripts with alternate exons that produce structural proteins, which are expressed in endothelia, epithelia (including bronchial epithelial cells), smooth muscle cells, and in nervous tissue. The major *CDH13* transcript contains 14 exons (Figure 3), encoding an open reading frame for a 713-amino acid polypeptide, cadherin 13, also known as T-cadherin, H-cadherin, or vascular adiponectin receptor. Previous GWAS have indicated that *CDH13* genetic variants may contribute to various phenotypes. The predicted molecular and cellular functions of cadherin 13 are congruent with some of the GWAS findings. The strongest and most consistent GWAS signals have been SNPs in the 5´ untranslated region or in intron 1 associated with circulating adiponectin levels, consistent with the molecular function of adiponectin binding (GO:0055100) (Chung et al. 2011; Dastani et al. 2012; Jee et al. 2010; Morisaki et al. 2012; Wu et al. 2010). *CDH13* SNPs have also been reported to be associated with body height (Okada et al. 2010) and with respiratory function (http://www.ncbi.nlm.nih.gov/projects/gapplusprev/sgap_plus.htm).

Cadherin 13 is one type of adiponectin-binding protein (Hug et al. 2004), among others such as adiponectin receptors (AdipoR1, AdipoR2) (Yamauchi et al. 2003) or calreticulin (Takemura et al. 2007), and might exert its role in respiratory health through adiponectin. Adiponectin—a 244 amino acid protein resembling collagen VII, X, and complement factor C1—has been identified as a potent and pleiotropic regulator of inflammation (Ohashi et al. 2012). Experimental evidence in mice demonstrated that cadherin 13 was required to mediate the protective effect of adiponectin on allergen-induced airway inflammation (Williams et al. 2012). In human studies, *CDH13* has been consistently identified by GWAS as a determinant of circulating adiponectin (Dastani et al. 2012), and serum adiponectin concentrations were positively associated with peak lung function in a prospective study of young healthy adults (Thyagarajan et al. 2010).

Adiponectin is secreted primarily by visceral adipocytes (Arita et al. 1999). Body composition and especially visceral adiposity have been associated with lower lung function and accelerated age-related decline (Rossi et al. 2011; Wehrmeister et al. 2012). We were among the first to report evidence of a modifying effect of obesity on the association of air pollution with lung function decline (Schikowski et al. 2013).

A strength of the current analysis is the detailed characterization of the cohort participants, as well as the cohort’s prospective design to specifically investigate longitudinally air pollution health effects. The fact that discovery and replication samples derive from the SAPALDIA cohort pool is a strength of this analysis. Both samples were recruited at the same time by the same field workers using the same standardized procedures. Nonetheless, larger studies in independent populations with different environmental and ethnic characteristics are needed to confirm the observed interactions between *CDH13* genetic variants and PM_10_. Genome-wide interactions between environmental exposures and genetic variants on complex health phenotypes form an active field of investigation, and novel methodologies are being developed to address analytical challenges associated with this research (Ege et al. 2011; Gauderman et al. 2013; Hutter et al. 2013; Sohns et al. 2013). Recently, a genome-wide analysis investigating genetic modifiers of associations between occupational exposures and lung function combined the GWIS approach with an *in silico* pathway analysis that indicated the involvement of inflammatory pathways (Liao et al. 2013).

There are several limitations, in addition to low power, in the present study. First, less than half of all baseline cohort participants were included in the analysis, leaving room for potential bias. Second, given the pleiotropic health outcomes associated with *CDH13* in previous GWAS, it is conceivable that modification of the air pollution–lung function association reflected an underlying susceptibility caused by health conditions associated with *CDH13* genotypes, such as cardiovascular phenotypes, rather than a causal interaction between the genotypes and PM_10_. In the absence of measured adiponectin levels we cannot verify whether the apparent modifying effect of *CDH13* is mediated through this adipokine. Genetic variants of the adiponectin precursor protein encoded by the gene *ADIPOQ* were by definition included in our GWIS analysis. We looked up the interactions between PM_10_ exposure and nine haplotype tagging variants of the *ADIPOQ* gene (Peters et al. 2013). None of these SNPs showed significant interactions (*p*_int_ = 0.07 to *p*_int_ = 0.95; for regional association plot of the *ADIPOQ* locus, see Supplemental Material, Figure S3). The *CDH13* SNPs associated with adiponectin circulating level (rs3865188, rs4783244, rs12051272) (Chung et al. 2011; Dastani et al. 2012; Jee et al. 2010; Morisaki et al. 2012; Wu et al. 2010) were in very low LD (*r*^2^ < 0.1) and thus contained in a different haplotype block than the *CDH13* SNPs interacting with PM_10_ (Figure 3). Nevertheless, rs3865188 previously associated with adiponectin (Jee et al. 2010; Wu et al. 2010) interacted with PM_10_ at a nominal *p*-value of 0.06 in our study.

Because we focused *a priori* on FEF_25–75%_ decline for this GWIS, we did not evaluate interactions between PM_10_ and previously identified GWAS signals on associations with FEV_1_ or FVC (Artigas et al. 2011; Hancock et al. 2010, 2012; Obeidat et al. 2011; Repapi et al. 2010; Wilk et al. 2009). Another limitation is that although asthmatic subjects appear to be more vulnerable to effects of air pollution exposure (Trasande and Thurston 2005), we restricted the current analyses to nonasthmatics. This sample restriction was based on our previous finding of an extended heterogeneity in the GWAS-identified determinants of lung function decline of healthy individuals compared with asthmatics (Imboden et al. 2012). Finally, comparing genotype-stratified analyses in the SAPALDIA cohort, the size of effect modification by the *CDH13* SNPs reported here on the PM_10_–FEF_25–75%_ association is substantially smaller than the size of effect modification by *SERPINA1* genotypes (underlying intermediate alpha 1 antitrypsin deficiency) on the association of FEF_25–75%_ decline with occupational exposure to vapors, dusts, gases, and fumes (Mehta et al. 2012). This is not unexpected for genome-wide signals of unknown functional relevance. The limitation of genome-wide signals with regard to clinical utility is a well-known problem of GWAS.

In conclusion, the mechanistic link between adiponectin (its modulating action on inflammatory processes systemically and locally in the lung) and cadherin 13 (its sequestering action on circulating adiponectin levels) make our GWIS finding, *CDH13*, a biologically plausible candidate gene for modifying the air pollution exposure effect. Follow-up studies need to confirm the observed interaction with *CDH13* SNPs and must assess whether the finding is related to recent evidence on the modifying effect of obesity on the association between PM_10_ and decline in lung function.

## Supplemental Material

(1.7 MB) PDF
