# Supplementary material for: Modification of the Association between PM10 and Lung Function Decline by Cadherin 13 Polymorphisms in the SAPALDIA Cohort: A Genome-Wide Interaction Analysis
Source: Environ Health Perspect. 2014 Aug 15;123(1):72–9. doi: 10.1289/ehp.1307398 (PMC4286270; doi:10.1289/ehp.1307398)
Supplement: (1.7 MB) PDF [file ehp.1307398.s001.508.pdf]

Supplemental Material

Modification of the Association between PM_{10} and Lung Function Decline by Cadherin 13 Polymorphisms in the SAPALDIA Cohort: A Genome-Wide Interaction Analysis

Medea Imboden, Ashish Kumar, Ivan Curjuric, Martin Adam, Gian Andri Thun, Margot Haun, Ming-Yi Tsai, Marco Pons, Robert Bettschart, Alexander Turk, Thierry Rochat, Nino Künzli, Christian Schindler, Florian Kronenberg, and Nicole M. Probst-Hensch

Table of Contents **Page**

Table S1. Top 1,000 GWIS association signals for adjusted interaction with cumulative PM_{10} on FEF_{25-75}, in SAPALDIA non-asthmatic discovery sample 2

Table S2. *CDH13* top hits identified for adjusted interaction with cumulative PM_{10} on FEF_{25-75} and their genome-wide ranking in GWIS analyses with cumulative PM_{10} on annual decline in other lung function phenotypes, in SAPALDIA non-asthmatic discovery sample 3

Table S3. Adjusted association of cumulative PM_{10} during eleven-year-follow-up on annual decline in FEF_{25-75}, by genotype strata, the SAPLADIA cohort. PM_{10} effect was significant in participants who carried the major allele homozygously 5

Table S4. GWIS results observed in the SAPALDIA discovery sample for *CDH13* genetic variants identified in previous GWAS reports 6

Figure S1. Higher resolution regional association plot (200 kb window) of chr16q23.3 and linkage disequilibrium structure underlying the same chromosomal window centred on GWIS top hit rs2325934 8

Figure S2. Derived haplotypes in the SAPALDIA discovery sample of the 200 kb-genomic region centred on the GWIS top signal, rs2325934 9

Figure S3. Regional association plot showing a 200 kb chromosomal window containing the *ADIPOQ* gene 10

References 11

Table S1. Top 1,000 GWIS association signals for adjusted[a] interaction with cumulative PM_{10} on FEF_{25-75}, in SAPALDIA non-asthmatic discovery sample[b]. The GWIS association results were sorted according to the *P*-values for gene x environment interaction effects (P_{int}) and the top 1,000 hits are are listed in the separate Excel file Supplemental Material, Table S1. The following terminology was used to report the calculated *P*-values of the genetic effects related to the gene marginal (P_{main}), the gene-by-environment (P_{int}), and the joint (P_{joint}) effects, referring to their respective null hypothesis of gene marginal (β_{main}=0), the gene-by-environment (β_{int}=0), and the joint (β_{main}=0 and β_{int}=0) effects.

Footnote to Table S1:
[a]GWIS was adjusted for study center, age, gender, height, smoking status, packyears at baseline and during follow-up, weight at baseline, weight change during follow-up, interaction between baseline weight and weight change, seasonal effects of time point of baseline and follow-up examination date (sine and cosine function of day of examination) and population stratification components. [b]Cohort participants with self-report of asthma history had been excluded from the analysis. Discovery sample size was N=763.

Table S2. *CDH13* top hits identified for adjusted[a] interaction with cumulative PM_{10} on FEF_{25-75} and their genome-wide ranking in GWIS analyses with cumulative PM_{10} on annual decline in other lung function phenotypes, in SAPALDIA non-asthmatic discovery sample[b]. P_{joint} tests were not performed for these models because they did not appear to identify additional SNPs based on the discovery GWIS analysis of FEF_{25-75}.

dbSNPID	Chr[c]	Position	Minor allele frequency	P_{main}[d]	P_{int}[d]	Genome-wide ranking: P_{main}[d]	Genome-wide ranking: P_{int}[d]
**Annual decline in FEV_1**							
rs2325934	16	81900000	9.62%	0.004	0.008	13785	24229
rs17282232	16	81905824	11.02%	0.002	0.005	6921	17067
rs10514582	16	81910432	8.40%	0.037	0.059	98971	148027
rs10514580	16	81910872	9.62%	0.005	0.012	17103	35475
rs16960234	16	81913512	9.86%	0.003	0.006	8768	17442
rs12325503	16	81917248	11.01%	0.001	0.001	1999	4568
rs10514578	16	81917312	11.00%	0.001	0.001	2026	4586
rs17210599	16	81918568	9.81%	0.002	0.004	7299	13182
rs10514575	16	81931320	9.71%	0.003	0.006	11089	18554
rs17211371	16	81933040	9.87%	0.004	0.008	14946	23995
rs1424168	16	81935600	10.03%	0.006	0.01	19729	30558
rs17211581	16	81937240	10.05%	0.007	0.011	21000	32111
rs17284098	16	81947576	12.61%	0.02	0.024	55827	65284
rs17284265	16	81949792	11.95%	0.012	0.015	35492	42089
rs17284390	16	81954784	11.69%	0.009	0.01	27749	29794
rs17212165	16	81955688	11.69%	0.009	0.009	27046	28084
rs11643197	16	81964792	12.87%	0.001	0.001	4832	4823
**Annual decline in FEV_1/FVC**							
rs2325934	16	81900000	9.59%	6.41E-08	1.99E-06	1	8
rs17282232	16	81905824	11.12%	3.14E-06	5.19E-05	22	302
rs10514582	16	81910432	8.40%	2.37E-06	4.39E-05	18	239
rs10514580	16	81910872	9.59%	1.95E-07	6.80E-06	6	36
rs16960234	16	81913512	9.84%	2.04E-07	6.82E-06	8	38
rs12325503	16	81917248	11.12%	8.75E-06	1.26E-04	38	727
rs10514578	16	81917312	11.11%	8.20E-06	1.16E-04	37	688
rs17210599	16	81918568	9.78%	1.94E-07	5.46E-06	5	28
rs10514575	16	81931320	9.62%	1.30E-07	2.34E-06	2	10
rs17211371	16	81933040	9.78%	1.62E-07	2.39E-06	3	11
rs1424168	16	81935600	9.94%	2.28E-07	3.05E-06	9	14
rs17211581	16	81937240	9.97%	2.54E-07	3.45E-06	10	16
rs17284098	16	81947576	12.48%	1.13E-06	9.78E-06	15	48
rs17284265	16	81949792	11.81%	3.27E-07	3.09E-06	11	15

dbSNPID	Chr[c]	Position	Minor allele frequency	P_{main}[d]	P_{int}[d]	Genome-wide ranking: P_{main}[d]	Genome-wide ranking: P_{int}[d]
rs17284390	16	81954784	11.54%	1.81E-07	1.81E-06	4	6
rs17212165	16	81955688	11.56%	1.99E-07	1.49E-06	7	5
rs11643197	16	81964792	12.91%	5.52E-06	2.21E-05	26	103
**Annual decline in FEF_{25-75}/FVC**							
rs2325934	16	81900000	9.59%	7.67E-08	1.47E-06	1	7
rs17282232	16	81905824	11.12%	4.90E-06	5.48E-05	47	338
rs10514582	16	81910432	8.40%	2.05E-07	4.84E-06	2	35
rs10514580	16	81910872	9.59%	2.24E-07	4.60E-06	3	34
rs16960234	16	81913512	9.84%	2.73E-07	5.50E-06	5	41
rs12325503	16	81917248	11.12%	2.13E-05	2.14E-04	99	1293
rs10514578	16	81917312	11.11%	2.04E-05	2.00E-04	96	1220
rs17210599	16	81918568	9.78%	3.63E-07	6.00E-06	19	43
rs10514575	16	81931320	9.62%	3.55E-07	3.69E-06	18	29
rs17211371	16	81933040	9.78%	4.72E-07	3.95E-06	20	32
rs1424168	16	81935600	9.94%	7.85E-07	5.36E-06	24	40
rs17211581	16	81937240	9.97%	9.23E-07	6.08E-06	25	44
rs17284098	16	81947576	12.48%	4.18E-06	1.93E-05	44	123
rs17284265	16	81949792	11.81%	1.38E-06	7.89E-06	31	54
rs17284390	16	81954784	11.54%	6.86E-07	4.09E-06	22	33
rs17212165	16	81955688	11.56%	7.50E-07	3.81E-06	23	30
rs11643197	16	81964792	12.91%	2.91E-05	8.99E-05	150	555

FEV_1: forced expiratory volume in the first second. FEV_1/FVC: Ratio between forced expiratory volume in the first second and forced vital capacity. FEF_{25-75}/FVC: Ratio between forced mid expiratory flow and forced vital capacity.

[a]GWIS was adjusted for study center, age, gender, height, smoking status, packyears at baseline and during follow-up, weight at baseline, weight change during follow-up, interaction between baseline weight and weight change, seasonal effects of time point of baseline and follow-up examination date (sine and cosine function of day of examination) and population stratification components. [b]Cohort participants with self-report of asthma history had been excluded from the analysis. Discovery sample size was N=763. [c]Chr – Chromosome. [d]The calculated *P*-values of the genetic effects related to the gene marginal effect is abbreviated as P_{main} and the *P*-values of the gene-by-environment as P_{int}.

Table S3. Adjusted[a] association of cumulative PM_{10} during eleven-year-follow-up on annual decline in FEF_{25-75}, by genotype strata, the SAPLADIA cohort. PM_{10} effect was significant in participants who carried the major allele homozygously. For reasons of sample size, we combined the heterozygous group with that of the homozygous minor allele group. Study sample for this table is the discovery and replication sample combined (N=4659).

**SNP genotype**	**N**	**Coefficient[b] (95% CI)**	***P*-value**
rs2325934[c]			
Homozygous major allele	3750	-0.1019 (-0.1915, -0.0122)	0.026
At least one minor allele	886	0.0741 (-0.1577, 0.3060)	0.531
rs1728409[d]			
Homozygous major allele	3568	-0.0803 (-0.1707, 0.0100)	0.081
At least one minor allele	1069	0.013 (-0.1980, 0.2240)	0.904

[a]Adjustments applied were the same as for the discovery GWIS including study center, age, gender, height, never smoking status, weight at baseline, weight change during follow-up, interaction between baseline weight and weight change, seasonal effects of time point of baseline and follow-up examination date (sine and cosine function of day of examination). No adjustment for population stratification was available. [b]Coefficient refers to the annual change in FEF_{25-75} [ml s^{-1}] per 1 $\mu g/m^3$ change in PM_{10} exposure. [c]For rs2325934: missing genotypes in discovery and replication sample (N=23). [d]For rs1728409: missing genotypes in discovery and replication sample (N=22).

Table S4. GWIS results observed in the SAPALDIA discovery sample for *CDH13* genetic variants identified in previous GWAS reports. Presented are the genetic effects of gene marginal[a] (P_{main}), of interaction[a] (P_{int}) and of the joint effect[a] (P_{joint}) of their interactions with cumulative PM_{10} exposure on annual decline in FEF_{25-75}. Next to the well-established association of CDH13 variants with circulating adiponectin levels (Chung et al. 2011; Dastani et al. 2012; Jee et al. 2010; Morisaki et al. 2012; Wu et al. 2010), less confirmed *CDH13* GWAS signals suggest a pleiotropic role of this protein. A number of neurologic and behavioural phenotypes, including attention deficit hyperactivity disorder (Lesch et al. 2008), depression (Terracciano et al. 2010a), cognitive performance (Cirulli et al. 2010), amphetamine response (Hart et al. 2012) and neuroleptic drug response (Adkins et al. 2011) or alcohol dependence (Treutlein et al. 2009) might be mediated by the cellular role of *CDH13* in neuron projection (GO:0043005). The predicted molecular function of calcium ion binding (GO:0005509) was supported by a GWAS finding of an association with level of Ca^{2+}-binding proteins (Benjamin et al. 2007). Other GWAS associations of CDH13 SNPs with cardiovascular phenotypes as blood pressure (Levy et al. 2007) and coronary artery disease (Wellcome Trust Case Control 2007) were less readily linked to predicted roles of cadherin 13.

dbSNP ID	Chr	Pos	MAF[b]	Position relative to *CDH13* gene	P_{main}	P_{int}	P_{joint}	GWAS-associated phenotypes	Reference
rs3865188	16	81208216	45.85%	-9693	0.09	0.063*	0.177	Adiponectin levels	Jee et al. 2010; Wu et al. 2010)
rs4783244	16	81219768	44.36%	1859	0.16	0.115	0.281	Adiponectin levels	Chung et al. 2011
rs12051272	16	81220792	0.63%	2883	0.32	0.285	0.554	Adiponectin levels	Dastani et al. 2012; Morisaki et al. 2012
rs10514556[c]	16	81276841	0	58932				Respiratory function, body weight	Fox et al. 2007; Wilk et al. 2007
rs11640875[d]	16	81278928	40.04%	61019	0.06	0.043*	0.127	Alcohol dependence	Treutlein et al. 2009
rs11640875[d]	16	81278928	40.04%	61019	0.06	0.043*	0.127	Myocardial infarction, alcohol dependence	dbGAP 2013
rs11646411	16	81304440	10.55%	86531	0.63	0.681	0.877	Attention deficit hyperactivity disorder	Lesch et al. 2008
rs12386026[c]	16	81375092	0	157183				Tuberculosis	Thye et al. 2010
rs12928678	16	81480080	29.26%	262171	0.56	0.465	0.724	Basophils count	dbGAP 2013
rs12597778	16	81551632	27.09%	333723	0.40	0.350	0.637	Myocardial infarction	dbGAP 2013
rs7404645	16	81578032	28.35%	360123	0.05	0.021*	0.045	Coronary artery disease	dbGAP 2013
rs9888896	16	81622904	29.02%	404995	0.07	0.037*	0.093	Coronary artery disease	dbGAP 2013
rs8055236	16	81769896	17.69%	551987	0.11	0.104	0.267	Coronary artery disease	Wellcome Trust Case Control 2007
rs17749270	16	81816392	14.47%	598483	0.53	0.588	0.799	Heart failure	dbGAP 2013
rs889723	16	81817560	37.43%	599651	0.95	0.966	0.944	Forced expiratory volume	dbGAP 2013
rs11150555	16	81827752	22.31%	609843	0.52	0.370	0.484	Attention deficit hyperactivity disorder	Lasky-Su et al. 2008
rs2194341	16	81837944	20.46%	620035	0.40	0.432	0.702	Tunica media thickness	dbGAP 2013
rs10514585	16	81841840	22.41%	623931	0.88	0.723	0.780	Depression	Terracciano et al. 2010
rs17195894	16	81877384	20.86%	659475	0.39	0.418	0.684	Body height	dbGAP 2013
rs6563898	16	81916280	31.14%	698371	0.63	0.653	0.890	Body height	dbGAP 2013

dbSNP ID	Chr	Pos	MAF[b]	Position relative to *CDH13* gene	P_{main}	P_{int}	P_{joint}	GWAS-associated phenotypes	Reference
rs10514576	16	81926144	11.89%	708235	0.71	0.672	0.910	Electrocardiography	Newton-Cheh et al. 2007
rs10514573	16	82011352	15.50%	793443	0.08	0.053*	0.142	Hippocampus structure	Seshadri et al. 2007
rs17216786	16	82044976	8.22%	827067	0.50	0.592	0.750	Clozapine response	Adkins et al. 2011
rs12919255	16	82093040	26.58%	875131	0.50	0.836	0.219	Potassium level	dbGAP 2013
rs4238691	16	82127384	42.80%	909475	0.08	0.105	0.224	Schizophrenia	dbGAP 2013
rs9930750	16	82146992	34.84%	929083	0.30	0.571	0.150	Schizophrenia	dbGAP 2013
rs4357934	16	82148720	34.81%	930811	0.31	0.593	0.157	Schizophrenia	dbGAP 2013
rs6563943[d]	16	82196832	39.69%	978923	0.12	0.103	0.264	Body height	Okada et al. 2010
rs6563943[d]	16	82196832	39.69%	978923	0.12	0.103	0.264	Waist circumference	dbGAP 2013
rs6563943[d]	16	82196832	39.69%	978923	0.12	0.103	0.264	Body mass index	dbGAP 2013
rs10514590	16	82206272	2.75%	988363	0.10	0.264	0.115	Calcium-binding protein level	Benjamin et al. 2007
rs8058532	16	82267168	45.61%	1049259	0.56	0.274	0.134	Amphetamine response	Hart et al. 2012
rs8059763	16	82283408	70.51%	1065499	0.76	0.695	0.905	Coronary artery disease	dbGAP 2013
rs8059763	16	82283408	70.51%	1065499	0.76	0.695	0.905	Coronary artery disease	dbGAP 2013
rs12446894	16	82285336	37.51%	1067427	0.77	0.900	0.849	Coronary artery disease	dbGAP 2013
rs12446894	16	82285336	37.51%	1067427	0.77	0.900	0.849	Coronary artery disease	dbGAP 2013
rs11861962	16	82292632	46.70%	1074723	0.36	0.349	0.642	Coronary artery disease	dbGAP 2013
rs11861962	16	82292632	46.70%	1074723	0.36	0.349	0.642	Coronary artery disease	dbGAP 2013
rs7198252	16	82294840	26.43%	1076931	0.77	0.720	0.921	Coronary artery disease	dbGAP 2013
rs7198252	16	82294840	26.43%	1076931	0.77	0.720	0.921	Coronary artery disease	dbGAP 2013
rs3784962	16	82314832	41.55%	1096923	0.76	0.824	0.928	Cognitive performance	Cirulli et al. 2010
rs3096277	16	82321704	18.99%	1103795	0.37	0.357	0.654	Blood pressure	Levy et al. 2007
rs9932947	16	82329440	37.02%	1111531	0.90	0.898	0.728	Heart failure	dbGAP 2013
rs10514597	16	82343264	1.40%	1125355	0.23	0.219	0.467	Blood pressure	Levy et al. 2007

Abbreviations: Chr: Chromosome; MAF: minor allele frequency; Pos – position (pb) (build36); P_{int}: P-value for interaction; P_{joint}: P-value for joint effect of SNP main and interaction of SNP with cumulative PM_{10}.

[a]Following terminology was used to report the results of the genetic effects related to the gene marginal (P_{main}), the gene-by-environment (P_{int}), and the joint (P_{joint}) effects referring to their respective null hypothesis of gene marginal (β_{main}=0), the gene-by-environment (β_{int}=0), and the joint (β_{main}=0 and β_{int}=0) effects. [b]Minor allele frequencies in SAPALDIA. [c]SNPs (rs10514556, rs12386026) are monomorphic in SAPALDIA. [d]Pleiotropic variants: rs11640875 associated with alcohol dependence and also with myocardial infarction. rs6563943 associated with body mass index, waist circumference and with body height.

**P*-value <0.1

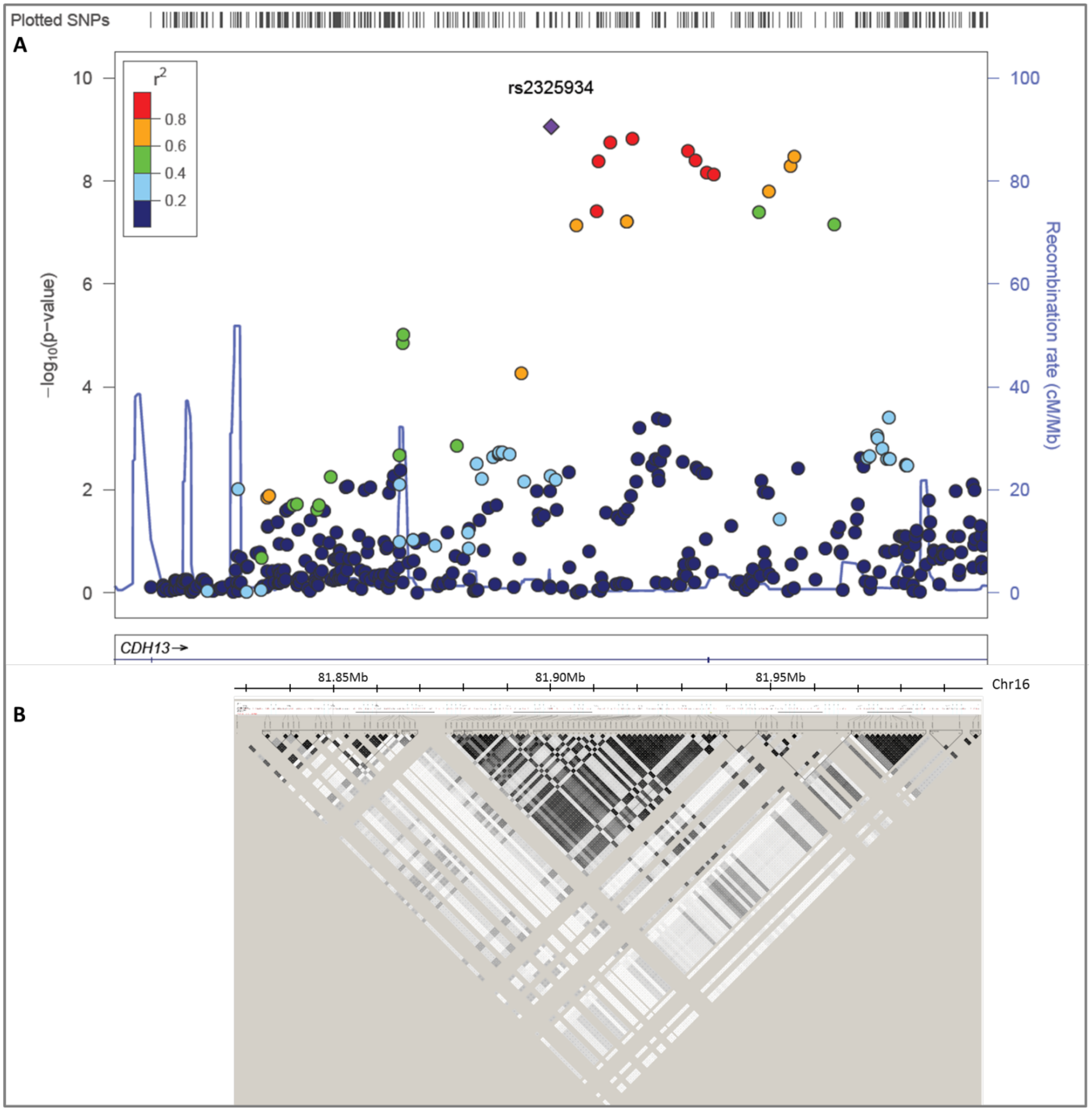

Figure S1. Higher resolution regional association plot (200 kb window) of chr16q23.3 and linkage disequilibrium structure underlying the same chromosomal window centred on GWIS top hit rs2325934. A - Regional association plot of a 200 kb window centred on rs2325934 in intron of *CDH13* gene; plotted are imputed SNPs. B - Linkage disequilibrium (LD) of the same 200 kb window; 105 SNPs were used to construct LD were selected for *P*-value for interaction of SNP with cumulative PM_{10} exposure ($P_{int} \leq 0.03$). LD grey scale depicting the LD metric r^2. Dark grey reflecting high LD; light grey low LD and white no LD.

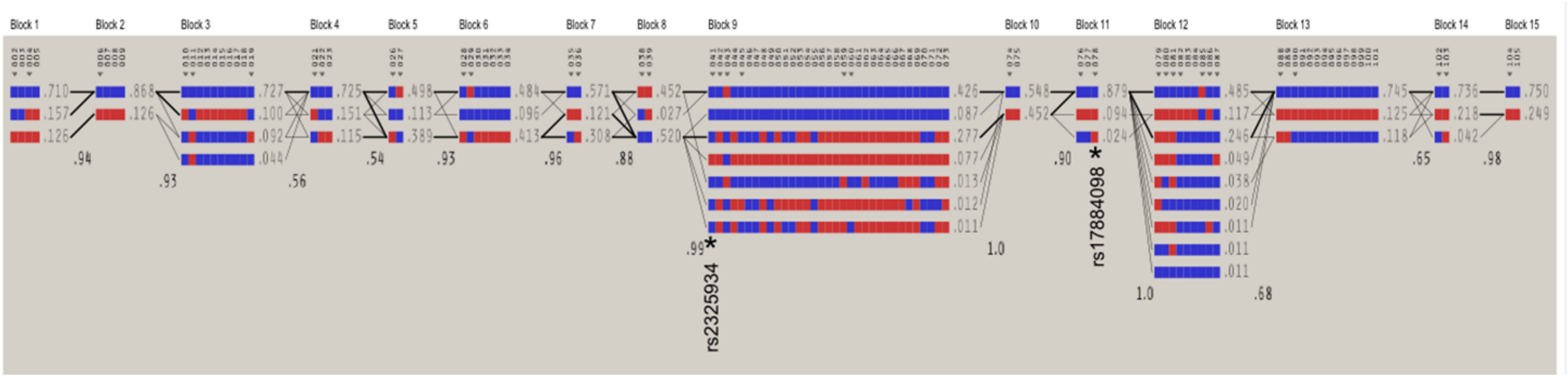

Figure S2. Derived haplotypes in the SAPALDIA discovery sample of the 200 kb-genomic region centred on the GWIS top signal, rs2325934. Shown are haplotypes in a 200b kb chromosomal window centred on rs2325934. The haplotpyes were built with imputed genotype data of the SAPALDIA discovery sample using the software, Haploview (Barrett et al. 2005). The SNPs are ordered equidistant above the haplotypes, with a tick beneath the SNP number indicating haplotype tagging SNPs. Two colors of the haplotypes are used to distinguish allele 1 from allele 2. Each haplotype is shown in a block with its population frequency connections from one block to the next. In the crossing areas, A value of multiallelic D', computed for the displayed alleles, is shown in the crossing areas, representing level of recombination between blocks. *Positions of the first, rs2325934, and second replication SNP, rs17884098.

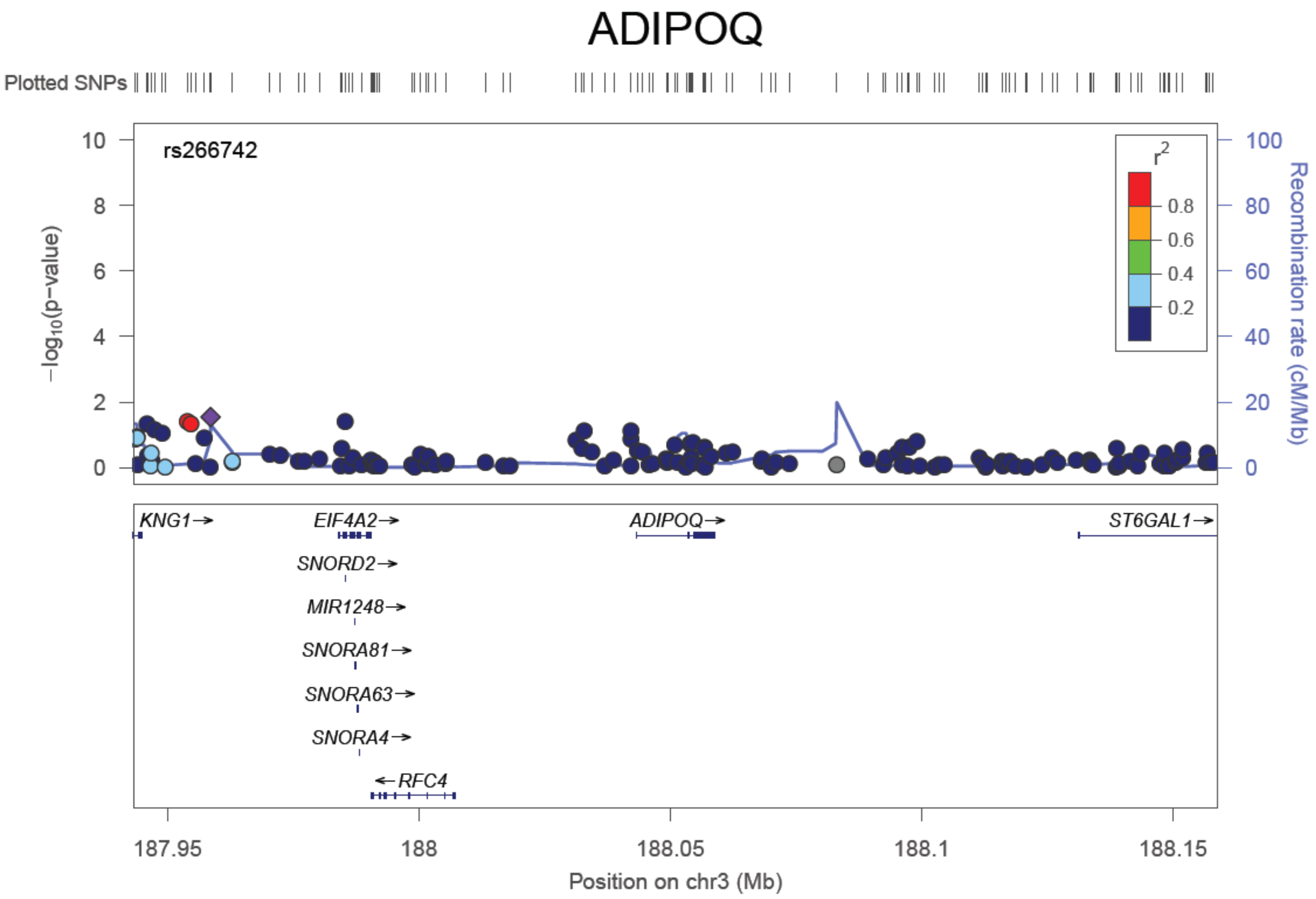

Figure S3. Regional association plot showing a 200 kb chromosomal window containing the *ADIPOQ* gene. The p-values of interaction between cumulative PM_{10} exposure and genetic variants on annual decline in $FEF_{25\text{-}75}$ are plotted, discovery sample (N=763) of the SAPALDIA cohort study. Shown is the regional association plot in a chromosomal window of 200 kb centered on the *ADIPOQ* gene. Negative log of the *P*-values are plotted on the Y-axis. Genomic coordinates (Mb) of the plotted SNPs refer to genome build 36/hg18 and dbSNP128 and are given on the X-axis. Linkage disequilibrium information refers to HapMap Phase II data of Caucasian samples. Recombination rate shown over this chromosomal window indicates recombination sites as vertical lines. The plot was generated using LocusZoom (Pruim et al. 2010). Genes in the genomic vicinity are EIF4A2 - Eukaryotic initiation factor 4A ; KNG1 - High molecular weight kininogen deficiency; MIR1248 - MicroRNA 1248; RFC4 - Replication factor C, Subunit 4; SNORA4 - Small nucleolar RNA SNORA4; SNORA63 - Small nucleolar RNA SNORA63 ; SNORA81 - Small nucleolar RNA, H/ACA box 81; SNORD2 - Small nucleolar RNA SNORD2; ST6GAL1 - ST6 Beta-galactosamide alpha-2,6-sialyltransferse 1.

References

Adkins DE, Aberg K, McClay JL, Bukszar J, Zhao Z, Jia P, et al. 2011. Genomewide pharmacogenomic study of metabolic side effects to antipsychotic drugs. Molecular psychiatry 16:321-332.

Barrett JC, Fry B, Maller J, Daly MJ. 2005. Haploview: Analysis and visualization of LD and haplotype maps. Bioinformatics 21:263-265.

Benjamin EJ, Dupuis J, Larson MG, Lunetta KL, Booth SL, Govindaraju DR, et al. 2007. Genome-wide association with select biomarker traits in the Framingham heart study. BMC medical genetics 8 Suppl 1:S11.

Chung CM, Lin TH, Chen JW, Leu HB, Yang HC, Ho HY, et al. 2011. A genome-wide association study reveals a quantitative trait locus of adiponectin on cdh13 that predicts cardiometabolic outcomes. Diabetes 60:2417-2423.

Cirulli ET, Kasperaviciute D, Attix DK, Need AC, Ge D, Gibson G, et al. 2010. Common genetic variation and performance on standardized cognitive tests. European journal of human genetics : EJHG 18:815-820.

Dastani Z, Hivert MF, Timpson N, Perry JR, Yuan X, Scott RA, et al. 2012. Novel loci for adiponectin levels and their influence on type 2 diabetes and metabolic traits: A multi-ethnic meta-analysis of 45,891 individuals. PLoS genetics 8:e1002607.

dbGAP. 2013. The database of genotypes and phenotypes (dbgap) developed to archive and distribute the results of studies that have investigated the interaction of genotype and phenotype. Association browser query: Cdh13. Available: http://www.ncbi.nlm.nih.gov/projects/gapplusprev/sgap_plus.htm [accessed 20 March 2013].

Fox CS, Heard-Costa N, Cupples LA, Dupuis J, Vasan RS, Atwood LD. 2007. Genome-wide association to body mass index and waist circumference: The framingham heart study 100k project. BMC medical genetics 8 Suppl 1:S18.

Hart AB, Engelhardt BE, Wardle MC, Sokoloff G, Stephens M, de Wit H, et al. 2012. Genome-wide association study of d-amphetamine response in healthy volunteers identifies putative associations, including cadherin 13 (cdh13). PloS one 7:e42646.

Jee SH, Sull JW, Lee JE, Shin C, Park J, Kimm H, et al. 2010. Adiponectin concentrations: A genome-wide association study. American journal of human genetics 87:545-552.

Lasky-Su J, Anney RJ, Neale BM, Franke B, Zhou K, Maller JB, et al. 2008. Genome-wide association scan of the time to onset of attention deficit hyperactivity disorder. American journal of medical genetics Part B, Neuropsychiatric genetics : the official publication of the International Society of Psychiatric Genetics 147B:1355-1358.

Lesch KP, Timmesfeld N, Renner TJ, Halperin R, Roser C, Nguyen TT, et al. 2008. Molecular genetics of adult adhd: Converging evidence from genome-wide association and extended pedigree linkage studies. Journal of neural transmission 115:1573-1585.

Levy D, Larson MG, Benjamin EJ, Newton-Cheh C, Wang TJ, Hwang SJ, et al. 2007. Framingham heart study 100k project: Genome-wide associations for blood pressure and arterial stiffness. BMC medical genetics 8 Suppl 1:S3.

Morisaki H, Yamanaka I, Iwai N, Miyamoto Y, Kokubo Y, Okamura T, et al. 2012. Cdh13 gene coding t-cadherin influences variations in plasma adiponectin levels in the Japanese population. Human mutation 33:402-410.

Newton-Cheh C, Guo CY, Wang TJ, O'Donnell C J, Levy D, Larson MG. 2007. Genome-wide association study of electrocardiographic and heart rate variability traits: The Framingham heart study. BMC medical genetics 8 Suppl 1:S7.

Okada Y, Kamatani Y, Takahashi A, Matsuda K, Hosono N, Ohmiya H, et al. 2010. A genome-wide association study in 19 633 Japanese subjects identified lhx3-qsox2 and igf1 as adult height loci. Human molecular genetics 19:2303-2312.

Pruim RJ, Welch RP, Sanna S, Teslovich TM, Chines PS, Gliedt TP, et al. 2010. Locuszoom: Regional visualization of genome-wide association scan results. Bioinformatics 26:2336-2337.

Seshadri S, DeStefano AL, Au R, Massaro JM, Beiser AS, Kelly-Hayes M, et al. 2007. Genetic correlates of brain aging on mri and cognitive test measures: A genome-wide association and linkage analysis in the Framingham study. BMC medical genetics 8 Suppl 1:S15.

Terracciano A, Tanaka T, Sutin AR, Sanna S, Deiana B, Lai S, et al. 2010. Genome-wide association scan of trait depression. Biological psychiatry 68:811-817.

Thye T, Vannberg FO, Wong SH, Owusu-Dabo E, Osei I, Gyapong J, et al. 2010. Genome-wide association analyses identifies a susceptibility locus for tuberculosis on chromosome 18q11.2. Nature genetics 42:739-741.

Treutlein J, Cichon S, Ridinger M, Wodarz N, Soyka M, Zill P, et al. 2009. Genome-wide association study of alcohol dependence. Archives of general psychiatry 66:773-784.

Wellcome Trust Case Control C. 2007. Genome-wide association study of 14,000 cases of seven common diseases and 3,000 shared controls. Nature 447:661-678.

Wilk JB, Walter RE, Laramie JM, Gottlieb DJ, O'Connor GT. 2007. Framingham heart study genome-wide association: Results for pulmonary function measures. BMC medical genetics 8 Suppl 1:S8.

Wu Y, Li Y, Lange EM, Croteau-Chonka DC, Kuzawa CW, McDade TW, et al. 2010. Genome-wide association study for adiponectin levels in Filipino women identifies cdh13 and a novel uncommon haplotype at kng1-adipoq. Human molecular genetics 19:4955-4964.
